# Exploring the complex relationship between systemic lupus erythematosus and coronavirus disease 2019: genetic insights and potential protective mechanisms

**DOI:** 10.7189/jogh.15.04191

**Published:** 2025-07-11

**Authors:** Xiaoli Xu, An-Tian Chen, Yantao Ding, Tingting Zhu, Luyao Xia, Jingkai Xu, Liyue Sun, Lu Liu

**Affiliations:** 1 Department of Dermatology, The First Affiliated Hospital of Anhui Medical University, Hefei, Anhui, China; 2 Institute of Dermatology, Anhui Medical University, Hefei, Anhui, China; 3 Department of Cardiology, Peking Union Medical College Hospital, Chinese Academy of Medical Sciences & Peking Union Medical College, Beijing, China; 4 Department of Dermatology, China-Japan Friendship Hospital, Beijing, China; 5 Department of Health Management Centre, Zhongshan Hospital, Fudan University, Shanghai, China; 6 Department of General Practice, Zhongshan Hospital, Fudan University, Shanghai, China

## Abstract

**Background:**

Severe coronavirus disease 2019 (COVID-19) and systemic lupus erythematosus (SLE) have been reported to share common gene loci, but the causal relationship between them remains controversial.

**Methods:**

We conducted a linkage disequilibrium score regression analysis to assess the genetic correlations between SLE and the two traits (infection and severity) of COVID-19 in European populations. Mendelian randomisation analysis was then performed to explore the causal effect of SLE on susceptibility to these traits in both European and East Asian data sets. Lastly, enrichment analysis and Protein-Protein Interactions analysis were used to identify key pathways and genes involved, providing insights into the possible mechanism underlying the complex relationship between SLE and COVID-19.

**Results:**

A significant genetic correlation was observed between SLE and COVID-19 severity (genetic correlation (rg) = 0.340, *P* = 0.001). However, no significant genetic correlation was found with COVID-19 infection. Mendelian randomisation analysis revealed a negative causal effect of SLE on both COVID-19 infection (odds ratio (OR) = 0.986; 95% confidence interval (CI) = 0.975–0.997, *P* = 0.009) and severity (OR = 0.955; 95% CI = 0.921–0.990, *P* = 0.012) in European populations, with similar findings replicated in East Asians. Notably, interleukin-6 (IL-6) and tumour necrosis factor were identified as hub cytokines connecting SLE to COVID-19 infection, while IL-6 and interleukin-10 (IL-10) were pivotal in connecting SLE to COVID-19 severity.

**Conclusions:**

This study reveals a potentially protective effect of SLE against COVID-19 infection and severity, with IL-6, tumour necrosis factor, and IL-10 playing key roles. Despite immunosuppressant use, SLE patients showed no increased risk of severe outcomes, likely due to their heightened caution in avoiding infection. These findings challenge common assumptions and highlight the need for further research.

Coronavirus disease 2019 (COVID-19) presents a multifaceted threat to the human health, impacting not only the respiratory system but also triggering systemic inflammatory responses. These responses can escalate into a cytokine storm, overwhelming the body’s defences and leading to severe complications such as acute respiratory distress syndrome [1]. In systemic lupus erythematosus (SLE), immune dysregulation disrupts immune homeostasis, causing the immune system to mistakenly attack the body's own tissues. This characteristic dysregulation in SLE raises important questions about how patients with this condition respond to COVID-19, especially considering the widespread use of immunosuppressive therapies in their management [2].

Recent studies investigating the impact of SLE on COVID-19 have yielded conflicting results. On one hand, SLE patients are thought to be at heightened risk for COVID-19 due to their compromised immune systems and frequent use of immunosuppressive treatments, which could theoretically impair their ability to mount an effective defence against infections [3–6].On the other hand, some reports have observed lower-than- expected infection rates and severe outcomes among SLE patients, prompting speculation that certain medications used in SLE may confer a protective effect against COVID-19 severity [7–11]. These discrepancies underscore the complexity of the interplay between SLE and COVID-19, highlighting the need for further investigation into this relationship and its clinical implications.

Particularly, due to substantial confounding in previous observational studies and meta-analyses leading to inconsistent results, this study applies mendelian randomisation (MR) to clarify potential genetic links between SLE and COVID-19. The MR offers a robust method to clarify causal relationships by minimising bias from reverse causation and confounding variables [12]. Leveraging this approach, we conducted a two-sample MR analysis to explore the causal association between SLE and both COVID-19 susceptibility and severity. To further elucidate the molecular mechanisms underlying this relationship, we supplemented the MR analysis with pathway enrichment and Protein-Protein Interaction (PPI) analyses, aiming to identify critical genetic pathways and molecular targets at the intersection of SLE and COVID-19. This integrative approach provides valuable insights into the complex relationship between these two diseases and may inform future treatment strategies for SLE patients in the context of the COVID-19 pandemic.

## METHODS

### Study design

First, we performed linkage disequilibrium score regression (LDSC) analysis to assess the genetic association between SLE and COVID-19 outcomes (infection and severity). Next, we selected appropriate instrumental variables (IVs) for SLE and conducted a two-sample MR analysis to investigate the causal effect of SLE on COVID-19 infection and severity. To ensure the robustness of our findings, we performed sensitivity tests and inverse MR analysis. Additionally, we replicated the MR findings in East Asians. Finally, we conducted enrichment analysis and PPI analysis to identify key pathways and genes using shared genes between SLE and COVID-19 outcomes. The overall procedure was presented in the flow diagram (Figure S1 in the **Online Supplementary Document**).

### Primary GWAS data sources

The SLE summary data was obtained from a genome-wide association study (GWAS) involving 14 267 individuals of European ancestry (5201 SLE patients and 9066 controls) [13]. For COVID-19 data, we used data sets related to the infection and severity of COVID-19 respectively in European ancestry individuals from the COVID-19 Host Genetics Initiative, comprising a total of 3 072 110 volunteers (38 984 COVID-19 infection patients and 1 644 784 controls; 5101 COVID-19 severity patients and 1 383 241 controls) [14]. These specific GWAS data sets were selected because of their large size and representativeness among the available data sets at the time of the study, maximising statistical power and coverage of common variants. Details of these summary statistics are provided in Table S1 in the **Online Supplementary Document**.

### LDSC analysis

We used LDSC analysis to calculate the genetic correlations between SLE and two COVID-19 outcomes (infection and severity). The first trait (p1) was designated as SLE, and the second trait (p2) as COVID-19 infection and severity, respectively. We prepared the linkage disequilibrium (LD) Scores file of 1000 Genomes data in Europeans and converted the summary statistics into the required sum stats format. In this step, we needed to clean the summary statistics in two stages: filter out high-quality imputation single nucleotide polymorphisms (SNPs) according to HapMap3 SNPs and check allele consistency between the summary statistics and the LD Scores file. Lastly, we verified the mean chi^2^ value in the log file. The genetic correlations were expressed as ‘rg’, with a mean χ^2^ value above 1.02 indicating reliability [15].

### IVs for MR analysis

We extracted genome-wide significant SNPs (*P* < 5 × 10^−8^) with minor allele frequency greater than 0.01 from the summary statistics of the exposure data set. Second, to minimise the distortion of randomised allele distribution caused by LD, we singled out the independent SNPs by purifying the above extracted SNPs with a serious standard (squared correlation (r^2^)<0.001 and a window size = 10 000 kb). Additionally, we calculated the F-statistic for each IV on the website mRnd and excluded the weak IVs with values less than 10 [16]. Missing SNP absent in the outcome data set was substituted by a proxy SNP with LD r^2^≥0.80 between the proxy and the replaced one [17]. This r^2^ threshold is widely used to ensure strong proxy-index SNP correlation and minimise substitution bias. Finally, we discarded the ambiguous SNP which the alleles do not correspond in the exposure-outcome data set.

### Two-sample MR analysis

In the two-sample MR analysis, the inverse variance weighted (IVW) [18] method was principally used, with MR-Pleiotropy residual sum and outlier (MR-PRESSO) [19], MR-Egger regression [20], weighted median [21], as well as weighted mode [22] were adopted as complementary approaches. Causality was expressed as odds ratio (OR) with 95% confidential interval (CI) to estimate the effect of SLE on COVID-19 infection and severity.

### Sensitivity tests and reverse MR analysis

We assessed heterogeneity across IVs by Cochran's Q test in IVW and MR-Egger analysis. If the heterogeneity was not significant (*P*_heterogeneity_>0.05), we applied a fixed-effect model; otherwise, a random-effect model was used [23]. We then evaluated the horizontal pleiotropy using MR-Egger [20] and MR-PRESSO Global tests [19]. When the intercept was close to zero (*P*_pleiotropy_>0.05) and no outlier was found, we would draw a funnel plot to exhibit the results of the horizontal pleiotropy test [17]. Finally, we did the leave-one-out analysis and single SNP analysis to assess the disproportionate effect resulting from any individual SNP [17].

To explore the potential inverse causality, we conducted reverse MR analysis to assess whether COVID-19 infection or severity could affect SLE. The analysis procedures were the same as described above.

### Replication MR analysis in East Asian population

In the replication stage, we obtained SLE-associated SNPs (*P* < 5 × 10^−8^) from our previous study conducted in the Chinese Han population, which included 4199 cases and 8255 controls [24]. The manifestation of the summary data set is shown in Table S2 in the **Online Supplementary Document**. The summary statistics for COVID-19 infection (4459 cases and 36 121 controls) and severity (794 cases and 4862 controls) were sourced from the COVID19-hg GWAS meta-analyses round seven for East Asians [14]. Lastly, we used the same method as applied in Europeans to select IVs, conduct MR analysis, and perform sensitive analysis. Due to limited publicly available Asian data sets, we utilised our previously published SLE summary data to ensure ethnic consistency and diagnostic clarity. For COVID-19, we selected the largest and most representative data set available the conduct of this study.

### Data sources and analyses of overlapping genes

Gene lists associated with SLE, COVID-19 infection, and COVID-19 severity individually were extracted from the GeneCards database [25]. Genes were included if correlation scores exceeded median thresholds, except for SLE (threshold set at 4-fold median due to numerous associations). Final thresholds: SLE>5.80, COVID-19 infection >0.40, severity >0.30. Overlapping genes between SLE and COVID-19 infection, and between SLE and COVID-19 severity, were identified using the online tool Draw Venn Diagram. Next, we performed enrichment analyses using Gene ontology (GO) and Kyoto Encyclopedia of Genes and Genomes (KEGG) databases via bioinformatics resource DAVID [26,27]. To explore the key genes, we constructed a PPI network of the overlapping genes by the STRING database [28]. We visualised the PPI network using Cytoscape [29], identifying key genes via median thresholds of three centrality metrics: Degree (DC), Betweenness (BC), and Closeness (CC).

### Statistics and graphics

The LDSC analysis followed an online tutorial titled ‘Heritability and Genetic Correlation.’ The MR analysis was performed using the ‘TwoSampleMR’ and ‘MR-PRESSO’ packages in *R* version 4.2.3 (R Foundation for Statistical Computing, Vienna, Austria). Figures were generated using ‘TwoSampleMR’ and ‘ForestPlot’ in *R*. A *P*-value <0.05 (two-sided) was considered statistically significant.

## RESULTS

### Genetic association between SLE and COVID-19 severity in Europeans

In the LDSC analysis, the mean chi^2^ value for SLE (mean chi^2^ = 1.266), COVID-19 infection (mean chi^2^ = 1.048), and COVID-19 severity (mean chi^2^ = 1.052) were all greater than 1.02, allowing us to proceed with further analysis. As shown in **Table 1**, we found a significant genetic association between SLE and COVID-19 severity (rg (standard error (SE)) = 0.340 (0.107), Z-score = 3.193; *P* = 0.001). However, no significant correlation was observed between SLE and COVID-19 infection (rg (SE) = 0.144 (0.117), Z-score = 1.235; *P* = 0.217).

**Table 1:** LDSC analysis results of SLE related to COVID-19 infection and severity

Traits		Genetic correlation			
**p1**	**p2**	**rg**	**se**	**Z-score**	*P*-value*
SLE	COVID-19 infection	0.144	0.117	1.235	0.217
SLE	COVID-19 severity	0.340	0.107	3.193	0.001

LDSC – Linkage disequilibrium score regression, rg –genetic correlation, se – standard error, SLE – systemic lupus erythematosus

*P-value was considered statistically significant if <0.05 (two sides).

### IVs for COVID-19 infection and severity in Europeans

We extracted 44 SNPs as IVs from the exposure SLE data set. Nevertheless, four SNPs were unavailable in the COVID-19 infection data set and three SNPs were missing from the COVID-19 severity data set. To address this, we replaced them with three and two suitable proxy SNPs, respectively. In addition, one ambiguous SNP was excluded from the SLE-COVID-19 infection analysis (Table S3–4 in the **Online Supplementary Document****)**. Finally, we tested the causal relationship using 42 IVs for COVID-19 infection and 43 IVs for COVID-19 severity.

### MR results of SLE impact on the COVID-19 infection and severity in Europeans

**Figure 1:**
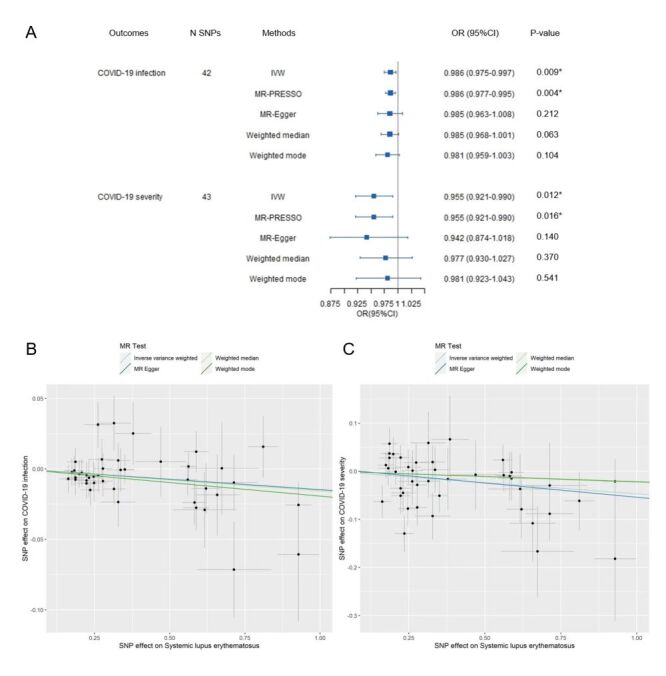
MR Results for the Effect of SLE on COVID-19 infection and severity in European populations. **Panel A.** Causal effects of SLE on COVID-19 infection and severity. **Panel B.** Scatter plot illustrating the impact of SLE on COVID-19 infection. **Panel C.** Scatter plot showing the relationship between SLE and COVID-19 severity. *P*-value <0.05. A *P*-value was considered statistically significant if <0.05 (two-tailed). CI – confidence interval, IVW – inverse variance weighted, MR – mendelian randomisation, MR-PRESSO – MR-Pleiotropy residual sum and outlier, N SNPs – number of SNPs, OR – odds ratio, SLE – systemic lupus erythematosus, SNP – single nucleotide polymorphism.

The results of IVW analysis indicated a negative association between SLE and COVID-19 infection (OR = 0.986; 95% CI = 0.975–0.997, *P* = 0.009), further validated by MR-PRESSO (OR = 0.986; 95% CI = 0.977–0.995, *P* = 0.004). Similarly, SLE exhibited an inverse correlation with COVID-19 severity in both IVW (OR = 0.955; 95% CI = 0.921–0.990, *P* = 0.012) and MR-PRESSO (OR = 0.955; 95% CI = 0.921–0.990, *P* = 0.016). Although other MR modes did not show statistically significant associations, the ORs were consistently below one (**Figure 1**, Panels A–C).

### Confirming the robustness and stability of MR results in Europeans

The *P*-values for heterogeneity in IVW and MR-Egger tests were both greater than 0.05, indicating no significant heterogeneity among the IVs, justifying the use of a fixed-effect model. The *P*-values for MR-Egger and MR-PRESSO global tests were greater than 0.05, showing no evidence of horizontal pleiotropy (Table S5–6 in the **Online Supplementary Document**). Symmetrical funnel plots further confirmed the stability of IVs. Leave-one-out analysis and single SNP analysis indicated no individual SNP disproportionately affected the results (Figure S2 in the **Online Supplementary Document**).

In the reverse MR analysis, we used three SNPs for COVID-19 infection and five SNPs for COVID-19 severity as IVs, respectively. No significant correlation was found, suggesting a unidirectional protective effect of SLE against both COVID-19 infection and severity (Figure S3 in the **Online Supplementary Document**).

### Replication study in East Asians

We replicated the MR analysis in East Asians. As shown in **Table 2**, the findings of IVW analysis confirmed the negative association between SLE and COVID-19 infection. However, the association between SLE and COVID-19 severity did not reach statistical significance, yet the OR remained below one. This implied that the direction of the effect in East Asians was consistent with the findings in Europeans.

**Table 2:** IVW analysis results of MR in East Asians

Outcomes	OR (95% CI)	N SNPs	*P*-value*	P_heterogeneity_
COVID-19 infection	0.946 (0.907–0.987)	15	0.011	0.900
COVID-19 severity	0.930 (0.817–1.058)	14	0.271	0.414

CI – confidence interval, IVW – Inverse variance weighted, MR – mendelian randomisation, N SNPs – number of single-nucleotide polymorphisms, OR – odds ratio

*P-value was considered statistically significant if <0.05 (two sides).

### Key pathways and hub genes in the relationship between SLE and COVID-19

**Figure 2:**
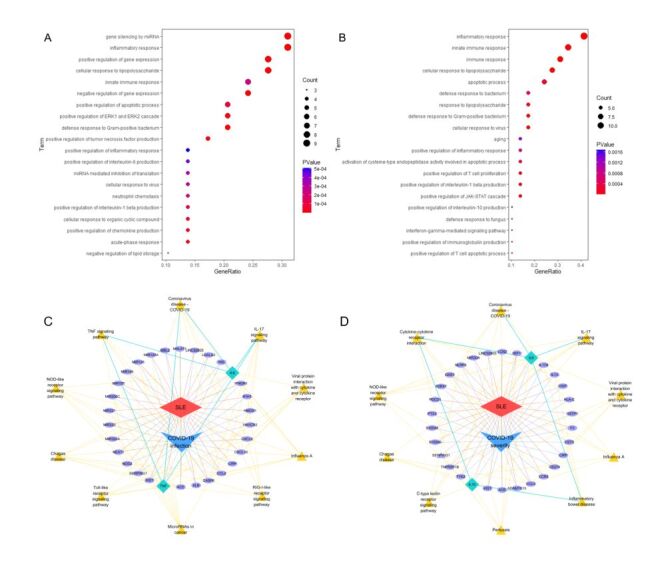
Enrichment analyses of overlapping genes between SLE and COVID-19 outcomes. **Panel A.** GO enrichment analysis for overlapping genes between SLE and COVID-19 infection. **Panel B.** GO enrichment analysis for overlapping genes between SLE and COVID-19 severity. **Panel C.** KEGG pathway enrichment analysis for common genes associated with SLE and COVID-19 infection. **Panel D.** KEGG pathway enrichment analysis for common genes linked to SLE and COVID-19 severity. In Panel C and Panel D, disease-related genes are displayed in purple oval shapes, with red lines connecting genes associated with SLE and blue lines connecting genes related to COVID-19. KEGG pathways are represented by yellow triangular shapes, linked by yellow lines to enriched genes. To highlight key genes identified in this study (IL6, TNF, IL10), these genes are specifically emphasised using bright green diamond-shaped frames, with bright green lines illustrating their associations with enriched pathways. GO – Gene ontology, IL-6 – Interleukin-6, IL-10 – Interleukin-10, KEGG – Kyoto Encyclopaedia of Genes and Genomes, SLE – systemic lupus erythematosus, TNF – tumour necrosis factor.

Based on the relevance scores, we identified a total of 587 genes associated with SLE, 153 genes related to COVID-19 infection, and 81 genes linked to COVID-19 severity. Among these, 30 genes overlapped between SLE and COVID-19 infection, and 30 genes overlapped between SLE and COVID-19 severity (Figure S4 in the **Online Supplementary Document**). The results of GO and KEGG enrichment analyses of these overlapping genes revealed significantly enriched pathways related to immune response, inflammatory process, and cellular response to viruses (Table S7–8 in the **Online Supplementary Document**). Notably, the positive regulation of interleukin-6 (IL-6) production, tumour necrosis factor (TNF) signalling pathways, and the NOD-like receptor signalling pathway were significantly enriched among the shared genes between SLE and COVID-19 infection (**Figure 2**, Panel A and Panel C). For genes overlapping between SLE and COVID-19 severity, enrichment was observed in pathways related to interleukin-10 (IL-10) production, cytokine-cytokine receptor signalling, and the NOD-like receptor signalling pathway (**Figure 2**, Panel B and Panel D).

**Figure 3:**
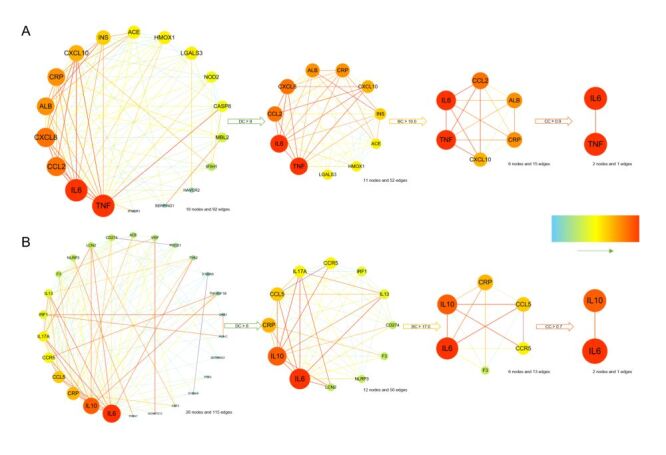
PPI network results. **Panel A.** PPI network illustrating the connection between SLE and COVID-19 infection. **Panel B.** PPI network demonstrating the linkage between SLE and COVID-19 severity. The direction of the emerald-hued arrows indicates a gradient, transitioning from light to dark from left to right. The size and depth of the circles increase accordingly, reflecting higher degree values for the respective genes and greater interaction magnitude within the PPI network. BC – betweenness centrality, CC – closeness centrality, DC – degree centrality, IL-6 – Interleukin-6, IL-10 – Interleukin-10, PPI – Protein-Protein Interactions, SLE – systemic lupus erythematosus, TNF – tumour necrosis factor.

In PPI network analysis using the STRING database, we identified 18 genes involved in the interaction between SLE and COVID-19 infection. and 27 genes related to the interaction between SLE and COVID-19 severity. One gene was excluded due to a lack of identified interactions between SLE and COVID-19 severity. Interestingly, IL-6 and TNF were identified as hub genes linking SLE to COVID-19 infection (DC>9.0, BC>10.0, and CC>0.9) (**Figure 3**, Panel A). For SLE and COVID-19 severity, IL-6 and IL-10 emerged as key genes (DC>8.0, BC>17.0, and CC>0.7) (**Figure 3**, Panel B).

## DISCUSSION

In this study, LDSC analysis revealed a significant genetic correlation between SLE and COVID-19 severity. Our MR analysis indicated that SLE patients had a reduced risk of both contracting COVID-19 and progressing to severe disease if infected, particularly in Europeans. These findings align with previous research suggesting that certain SLE risk alleles may offer protection against severe COVID-19 outcomes [30]. Comprehensive sensitivity analyses and reverse MR further reinforced the robustness of these conclusions. In the East Asian cohort, we replicated the causal link between SLE and COVID-19 infection. Although the association with COVID-19 severity was not statistically significant – likely due to smaller sample size – a similar protective trend (OR<1) matched European results, supporting cross-ethnic consistency. The results of LDSC analysis in Europeans did not detect significant genetic correlation, differing from MR outcomes and underscoring that genetic correlation does not equate causation. This phenomenon could be attributable to that LDSC evaluates overall shared genetic architecture, potentially missing weak signals from specific causal loci [15]. In contrast, MR specifically employs robust instrumental variables (SNPs with *P* < 5 × 10^−8^), capturing causal relationships independent of extensive genetic overlap [31,32]. Accordingly, in this study, we applied two-sample MR analysis to bridge the gap left by correlation analyses and to further investigate whether SLE has a direct causal influence on COVID-19 susceptibility and severity.

To explore the underlying mechanisms of this protective association, we conducted enrichment analyses focusing on genes shared between SLE and COVID-19 infection, as well as those linked to COVID-19 severity. The results emphasised the interleukin-17 signalling pathway and the COVID-19 immune response as key factors contributing to both preventing infection and reducing severity in SLE patients [33]. Additionally, distinct pathways were identified, providing insights into how SLE may confer protection against COVID-19 onset and progression to severe disease. For example, the regulation of IL-6 production and TNF signalling were intricately linked to infection, while IL-10 was associated with reduced severity. The results of PPI analysis supported these findings, highlighting the central roles of IL-6 and TNF in COVID-19 infection, with IL-10 emerging as a critical mediator of disease severity.

Contrary to initial assumptions, our results suggested a complex and potentially protective relationship between SLE and both COVID-19 infection and severity. Several factors may explain these findings:

o First, the reduced infection rates may stem from the stringent preventive measures taken by SLE patients. For example, by mid-April 2020, no SLE patients had been diagnosed with COVID-19 in Hong Kong, likely due to strict adherence to public health measures [34]. A survey of 62 Italian SLE patients during the COVID-19 peak in spring 2020 found no confirmed cases, with 95% strictly adhering to mask use and social distancing [35]. This observation suggests that practices such as social distancing, frequent handwashing, and wearing masks have likely played a critical role in mitigating the spread of COVID-19 among SLE patients.

o Second, the pathophysiology of SLE itself may offer protection. Patients with SLE commonly exhibit a type I interferon (IFN-I) gene signature characterised by persistently elevated antiviral gene expression even without infection, potentially offering rapid antiviral responses. Genetic variants linked to increased IFN-I pathway activity and higher SLE risk may enhance resistance to viruses like syndrome coronavirus 2 (SARS-CoV-2) [36], indicating a protective antiviral role for IFN-I hyperactivation in SLE. Moreover, several studies have reported reduced IFN-I responses in COVID-19 patients [37]. A recent study also showed that neutralising autoantibodies against IFN-I were present in at least 10% of patients with severe COVID-19 [38]. Thus, the heightened IFN-I activity in SLE patients may contribute to more efficient viral clearance, reducing the risk of severe outcomes.

o Lastly, the widespread use of immunosuppressive treatments in SLE, particularly corticosteroids and biologics, raises concerns about increased susceptibility to infection. However, studies have suggested that these therapies may paradoxically provide protection against severe COVID-19 outcomes [7-10]. For instance, the RECOVERY trial demonstrated that dexamethasone reduced mortality in hospitalised COVID-19 patients requiring mechanical ventilation [39]. Although the Global Rheumatology Alliance reported that glucocorticoids doses >10 mg/d increased the likelihood of hospitalisation, guidelines recommend tapering to the lowest effective dose to minimise complications [40]. Furthermore, low-dose steroids have been linked to reduced mortality in critically ill COVID-19 patients, indicating a dose-dependent effect on outcomes [41]. Biologics have also been investigated as therapies to mitigate inflammation and reduce the risk of acute respiratory distress syndrome in COVID-19 patients [42]. Our results identify IL-6 and TNF as critical therapeutic targets against COVID-19 in SLE patients, whereas IL-10 relates closely to severe outcomes. The underlying mechanisms may involve that IL-6 and TNF-α drive cytokine storms in severe COVID-19 [9] and are elevated during active SLE phases, causing tissue damage in both lupus and viral infections [43]. Notably, IL-6 and TNF-α blockade therapies, such as the IL-6 receptor inhibitor tocilizumab, have effectively reduced mortality in severe COVID-19 patients [44]. Additionally, elevated serum angiotensin converting enzyme 2 levels, associated with increased TNF-α production [45], have been observed in COVID-19 patients [2,46], suggesting that anti-TNF-α therapies used in SLE could provide benefit in treating COVID-19, as reported in some studies [7–9]. Observational studies indicate that autoimmune patients on anti–TNF-α therapy have reduced severe COVID-19 incidence, aligning with our findings linking IL-6 and TNF-α to infection susceptibility in SLE [9]. As for IL-10, elevated in both SLE and severe COVID-19, promotes B-cell activation in lupus but also reflects immune regulation [47]. Our results associating IL-10 with lower COVID-19 severity suggest elevated IL-10 might suppress cytokine storms, protecting SLE patients – a compensatory mechanism also proposed in COVID-19 [48]. Thus, chronic IL-10 elevation in SLE may reduce inflammation during SARS-CoV-2 infection. The physiological roles of these cytokines may help explain why SLE patients, despite immunosuppressants use, are not at a higher risk for severe COVID-19 and may even benefit from the medications used to manage their autoimmune disease. Immunosuppressants commonly used in SLE may confound genetically inferred causal links. Our MR analysis, based on summary-level data, could not adjust for individual treatments; thus, the observed protective effect likely reflects combined disease and treatment influences, necessitating cautious interpretation.

Our findings, while intriguing, have several limitations. First, publicly available GWAS summary data could introduce biases due to differences in COVID-19 diagnostic criteria and case inclusion. Using summary-level data prevented adjustment for individual-level confounders, such as medication and demographics, thereby requiring careful interpretation regarding potential medication influences. Second, the small sample size of the East Asian replication cohort limited statistical power, warranting further validation in larger, diverse populations. Additionally, proxy SNP usage for missing SNPs could introduce biases. Lastly, our reverse MR showed no significant effect of COVID-19 on SLE, though unmeasured confounders (*e.g*. general health or immune traits) might mask subtle causal effects. Despite minimal pleiotropy identified through sensitivity analyses, residual confounding remains possible, thus, our conclusion that COVID-19 does not exacerbate SLE warrants cautious interpretation.

Given these limitations, translating genetic findings into clinical practice requires caution and strict ethical adherence. Although MR analyses suggest potential protective effects of SLE or its therapies against COVID-19, clinical decisions should not solely rely on genetic evidence. Adjustments in SLE treatment during pandemic require individualised balancing between infection risk and lupus flare potential. Current guidelines recommend maintaining baseline therapies and minimising glucocorticoid doses [40]. Our findings do not support proactively increasing immunosuppressants or discontinuing essential medications without clinical indications. While IL-6 (eg, tocilizumab) [44] and TNF-α inhibitors [9] show promise, they involve risks such as infections [49] and lupus-like syndromes [50]. The IL-10 targeting remains exploratory. Ultimately, clinical translation of genetic insights requires rigorous trials and ethical validation, positioning our results as scientific support rather than direct clinical guidance.

## CONCLUSIONS

This study elucidates the complex relationship between SLE and COVID-19, identifying intrinsic mechanisms that may protect SLE patients from both infection and severe disease progression. These findings also provide insights into potential therapeutic targets, including IL-6, TNF, and IL-10, for the prevention and management of COVID-19 in SLE patients. Our results challenge conventional assumptions and offer new perspectives on the interaction between SLE and COVID-19. Further research is needed to confirm these findings and explore additional therapeutic targets.

Additional material: Online Supplementary Document
