# Supplementary material for: Exploring the complex relationship between systemic lupus erythematosus and coronavirus disease 2019: genetic insights and potential protective mechanisms
Source: J Glob Health. 2025 Jul 11;15:04191. doi: 10.7189/jogh.15.04191 (PMC12247663; doi:10.7189/jogh.15.04191)
Supplement: Additional material: Online Supplementary Document [file jogh-15-04191-s001.pdf]

Supplement to: Xu X, Chen A, Ding Y, Zhu T, Xia L, Xu J, Sun L, Liu L. Exploring the complex relationship between systemic lupus erythematosus and coronavirus disease 2019: genetic insights and potential protective mechanisms. J Glob Health. 2025;15:04191.

Abbreviations

SLE	Systemic lupus erythematosus
COVID-19	Coronavirus disease 2019
MR	Mendelian randomization
IVs	Instrumental variables
LDSC	Linkage disequilibrium score regression
SNP	Single nucleotide polymorphism
N SNPs	Number of SNPs
MAF	Minor allele frequency
IVW	Inverse variance weighted
MR-PRESSO	MR-Pleiotropy residual sum and outlier
CHR	Chromosome
POS	Base pair position
A1	Effect allele
A2	Alternative allele
OR	Odds ratio
CI	Confidence interval
GO	Gene ontology
KEGG	Kyoto Encyclopedia of Genes and Genomes
GWAS	Genome-wide association study

Supplementary tables

Table S1. The summary data characteristics of SLE, COVID-19 infection and severity

Variables	PMID	Cases	Controls	Simple size	Population
Exposure					
SLE	26502338	5,201	9,066	14,267	European ancestry
Outcomes					
COVID-19 infection	32404885	38,984	1,644,784	1,683,768	European ancestry
COVID-19 severity	32404885	5,101	1,383,241	1,388,342	European ancestry

Table S2. The significant SNPs associated with SLE in East Asians

SNP	CHR	POS	A1	A2	MAF	Beta	SE	P-value
rs1234315	1	173209324	A	G	0.50	0.315	0.030	2.34E-26
rs2205960	1	173222336	A	C	0.35	0.378	0.032	2.53E-32
rs13385731	2	33476823	G	A	0.11	-0.357	0.045	1.25E-15
rs7574865	2	191099907	A	C	0.42	0.412	0.030	5.17E-42
rs10036748	5	151078585	G	A	0.20	-0.211	0.035	1.67E-09
rs548234	6	106120159	G	A	0.31	0.223	0.032	5.18E-12
rs2230926	6	137874929	C	A	0.07	0.542	0.064	1.37E-17
rs4917014	7	50266267	C	A	0.25	-0.329	0.033	2.75E-23
rs1167796	7	75543861	A	G	0.25	-0.186	0.033	2.12E-08
rs4728142	7	128933913	A	G	0.18	0.358	0.040	8.14E-19
rs7812879	8	11482672	A	G	0.18	-0.371	0.036	2.09E-24
rs2618479	8	11498312	A	G	0.20	-0.329	0.035	5.26E-21
rs2248932	8	11534141	G	A	0.21	-0.274	0.036	1.63E-14
rs1913517	10	48911009	A	G	0.33	0.215	0.031	7.22E-12
rs4639966	11	118702810	G	A	0.36	0.255	0.031	1.25E-16
rs6590330	11	128441164	A	G	0.41	0.315	0.030	1.77E-25
rs10847697	12	128814840	A	G	0.25	0.231	0.035	3.54E-11
rs1385374	12	128816149	A	G	0.25	0.231	0.034	1.77E-11
rs7197475	16	30631546	A	G	0.10	0.270	0.049	2.77E-08

rs463426	22	21454896	G	A	0.45	-0.248	0.030	1.48E-16
rs131654	22	21562901	G	A	0.41	-0.248	0.030	2.99E-16

Table S3. SNPs used as IVs for SLE in Europeans

SNP	CHR	POS	A1	A2	MAF	Beta	SE	P-value	F-statistic
rs6679677	1	114303808	A	C	0.10	0.336	0.046	4.55E-13	110.78
rs4661543	1	15229101	G	T	0.88	0.274	0.042	9.40E-11	110.78
rs10912578	1	173251856	G	A	0.72	-0.247	0.031	1.65E-15	111.57
rs17849501	1	183542323	T	C	0.06	0.811	0.050	1.81E-59	93.99
rs6671847	1	161478810	A	G	0.47	0.199	0.029	6.64E-12	115.58
rs4916215	1	173314540	T	C	0.75	0.223	0.034	5.07E-11	118.00
rs12094036	1	183558174	C	T	0.08	-0.329	0.058	1.37E-08	124.41
rs13019891	2	113829869	T	G	0.46	-0.562	0.029	1.65E-83	115.68
rs2573219	2	233288667	C	A	0.09	0.588	0.043	1.13E-42	97.66
rs10200680	2	223961877	T	C	0.14	-0.248	0.042	4.96E-09	117.23
rs268124	2	65654364	T	C	0.72	0.186	0.032	8.60E-09	116.54
rs2459611	2	191939187	T	C	0.89	0.261	0.045	7.62E-09	113.95
rs4274624	2	191958656	T	C	0.77	-0.560	0.033	9.73E-66	110.35
rs10048743	2	213890232	T	G	0.86	-0.231	0.041	2.04E-08	114.97
rs34703115	2	40282854	C	T	0.04	-0.616	0.105	4.08E-09	161.37
rs1464446	3	146601295	T	G	0.18	-0.329	0.040	2.79E-16	122.78
rs9852014	3	129084581	G	A	0.07	0.621	0.049	2.26E-36	99.79
rs13136219	4	102743687	T	C	0.36	-0.174	0.028	3.50E-10	106.53
rs1078324	5	149202268	A	C	0.05	-0.713	0.078	7.11E-20	134.49
rs4388254	5	133428601	T	C	0.07	0.378	0.060	3.71E-10	122.07
rs2431697	5	159879978	C	T	0.42	-0.223	0.029	2.60E-14	115.55
rs6889239	5	150457771	C	T	0.25	0.278	0.032	2.19E-18	109.61
rs389884	6	31940897	G	A	0.07	0.928	0.043	2.92E-102	87.69
rs9274357	6	32632457	T	C	0.19	0.457	0.035	1.28E-38	109.99
rs7768653	6	106574794	T	C	0.59	-0.207	0.030	3.11E-12	116.90
rs12524498	6	31444187	T	G	0.01	-0.673	0.121	2.48E-08	94.04

rs58721818	6	138243739	T	C	0.02	0.658	0.076	3.38E-18	83.60
rs150180633	6	31010047	T	C	0.02	0.928	0.069	2.66E-41	76.40
rs28361029	6	31220203	A	G	0.07	-0.386	0.061	3.27E-10	123.99
rs35000415	7	128585616	T	C	0.10	0.588	0.042	1.86E-45	99.09
rs2736332	8	11339965	C	G	0.26	0.278	0.032	4.83E-18	112.19
rs7823055	8	55511676	T	G	0.56	-0.351	0.029	1.64E-34	113.70
rs7899626	10	63825561	T	C	0.36	0.182	0.033	4.19E-08	127.44
rs7097397	10	50025396	A	G	0.39	-0.186	0.029	8.60E-11	111.88
rs58688157	11	625085	G	A	0.26	-0.223	0.034	2.97E-11	117.40
rs353608	11	35101738	G	A	0.54	0.186	0.028	2.93E-11	111.74
rs73050535	12	5012503	T	C	0.03	-0.713	0.124	9.11E-09	165.80
rs597808	12	111973358	G	A	0.51	-0.163	0.029	3.51E-08	117.85
rs1143679	16	31276811	A	G	0.13	0.582	0.040	5.03E-48	106.98
rs13332649	16	85966683	G	A	0.20	-0.315	0.038	5.43E-17	119.69
rs143123127	17	38007190	A	G	0.04	0.470	0.084	2.23E-08	129.82
rs35251378	19	10459969	A	G	0.28	-0.236	0.032	3.61E-13	116.14
rs73068668	19	55763262	A	G	0.10	-0.315	0.057	4.40E-08	136.78
rs3747093	22	21984379	A	G	0.20	0.262	0.035	2.88E-14	109.98

Table S4. The proxied and ambiguous SNPs of IVs in Europeans

Outcomes	Proxy replacement		Ambiguous SNP
	Target SNP	Proxy SNP	
COVID-19 infection	rs150180633	rs75407132	rs28361029
	rs28361029	rs73728467	
	rs143123127	rs8068894	
	rs9274357	NA	
COVID-19 severity	rs150180633	rs75407132	/
	rs143123127	rs113233720	
	rs9274357	NA	

Table S5. The heterogeneity test for the MR results in Europeans

Outcomes	IVW			MR-Egger		
	Q	Q_df	$P_{heterogeneity}$	Q	Q_df	$P_{heterogeneity}$
COVID-19 infection	30.141	41	0.894	30.140	40	0.871
COVID-19 severity	50.832	42	0.165	50.675	41	0.143

Table S6. The horizontal pleiotropy test for the MR results in Europeans

Outcomes	MR-Egger			MR-PRESSO	
	Intercept	SE	$P_{pleiotropy}$	RSSobs	$P_{pleiotropy}$
COVID-19 infection	2.16E-04	4.18E-03	0.976	31.710	0.909
COVID-19 severity	5.03E-03	1.42E-02	0.724	52.766	0.190

Table S7. The top 20 GO pathways and top 10 KEGG pathways enriched by genes overlapped between SLE and COVID-19 infection

Category	Term	PValue	Count	Genes
GO:0071222	cellular response to lipopolysaccharide	7.66E-09	8	CXCL10, IL6, MIR223, CXCL8, CCL2, NOD2, TNF, HAVCR2
GO:0006954	inflammatory response	1.10E-07	9	CRP, CXCL10, IL6, MIR223, CXCL8, MIR221, CCL2, TNF, HAVCR2
GO:0035195	gene silencing by miRNA	1.01E-06	9	MIR126, MIR223, MIR145, MIR155, MIR221, MIR320A, MIR200C, NEAT1, MIR125A
GO:0050830	defense response to Gram-positive bacterium	1.08E-06	6	CRP, IL6, NOD2, TNF, HAVCR2, MBL2
GO:0010629	negative regulation of gene expression	4.34E-06	7	ACE, CXCL8, MIR155, TNF, HAVCR2, MIR125A, INS
GO:0010628	positive regulation of gene expression	6.23E-06	8	CRP, IL6, MIR223, CXCL8, IFNGR1, TNF, MIR125A, INS
GO:0032760	positive regulation of tumor necrosis factor production	1.55E-05	5	IFIH1, IL6, IFNGR1, NOD2, HAVCR2

GO:0070374	positive regulation of ERK1 and ERK2 cascade	1.58E-05	6	MIR126, MIR221, CCL2, NOD2, TNF, HAVCR2
GO:0006953	acute-phase response	2.37E-05	4	CRP, IL6, MBL2, INS
GO:0032722	positive regulation of chemokine production	3.66E-05	4	IL6, HMOX1, TNF, HAVCR2
GO:0071407	cellular response to organic cyclic compound	3.91E-05	4	CASP8, CCL2, NOD2, TNF
GO:0010888	negative regulation of lipid storage	7.18E-05	3	CRP, IL6, TNF
GO:0043065	positive regulation of apoptotic process	9.40E-05	6	IL6, ACE, CASP8, MIR221, HMOX1, TNF
GO:0032731	positive regulation of interleukin-1 beta production	1.06E-04	4	IL6, CASP8, NOD2, TNF
GO:0030593	neutrophil chemotaxis	2.20E-04	4	LGALS3, CXCL10, CXCL8, CCL2
GO:0045087	innate immune response	2.27E-04	7	IFIH1, CRP, LGALS3, SERPING1, NOD2, HAVCR2, MBL2
GO:0098586	cellular response to virus	2.90E-04	4	IFIH1, CXCL10, IL6, IFNGR1
GO:0035278	miRNA mediated inhibition of translation	3.30E-04	4	MIR126, MIR145, MIR221, MIR200C
GO:0032755	positive regulation of interleukin-6 production	4.31E-04	4	IFIH1, IL6, NOD2, TNF
GO:0050729	positive regulation of inflammatory response	5.09E-04	4	MIR126, ACE, NEAT1, TNF
hsa05164	Influenza A	1.11E-07	8	IFIH1, CXCL10, IL6, CXCL8, CASP8, IFNGR1, CCL2, TNF
hsa05142	Chagas disease	1.24E-07	7	IL6, ACE, CXCL8, CASP8, IFNGR1, CCL2, TNF
hsa05171	Coronavirus disease - COVID-19	8.89E-07	8	IFIH1, CXCL10, IL6, ACE, CXCL8, CCL2, TNF, MBL2
hsa04657	IL-17 signaling pathway	2.68E-06	6	CXCL10, IL6, CXCL8, CASP8, CCL2, TNF
hsa05206	MicroRNAs in cancer	6.17E-06	8	MIR126, MIR223, MIR145, MIR155, MIR221, HMOX1, MIR200C, MIR125A
hsa04668	TNF signaling pathway	6.95E-06	6	CXCL10, IL6, CASP8, CCL2, NOD2, TNF

hsa04622	RIG-I-like receptor signaling pathway	2.44E-05	5	IFIH1, CXCL10, CXCL8, CASP8, TNF
hsa04621	NOD-like receptor signaling pathway	7.42E-05	6	IL6, CXCL8, CASP8, CCL2, NOD2, TNF
hsa04061	Viral protein interaction with cytokine and cytokine receptor	9.40E-05	5	CXCL10, IL6, CXCL8, CCL2, TNF
hsa04620	Toll-like receptor signaling pathway	1.10E-04	5	CXCL10, IL6, CXCL8, CASP8, TNF

Table S8. The top 20 GO pathways and top 10 KEGG pathways enriched by genes overlapped between SLE and COVID-19 severity

Category	Term	PValue	Count	Genes
GO:0006954	inflammatory response	8.07E-12	12	P2RX7, CRP, IL6, CCL5, IL13, NLRP3, PTX3, CCR5, TNFRSF1B, S100A9, S100A8, IL17A
GO:0071222	cellular response to lipopolysaccharide	7.66E-09	8	IL10, CD274, IL6, ADAMTS13, GSTP1, NLRP3, CCR5, TNFRSF1B
GO:0045087	innate immune response	1.44E-07	10	CRP, OAS1, LCN2, HLA-C, NLRP3, SERPING1, PTX3, S100A9, S100A8, IL17A
GO:0006955	immune response	3.45E-07	9	IL10, CD274, CCL5, IL13, HLA-C, TYK2, CCR5, TNFRSF1B, IL17A
GO:0098586	cellular response to virus	8.12E-06	5	IL6, OAS1, CCL5, NLRP3, TYK2
GO:0050830	defense response to Gram-positive bacterium	3.47E-05	5	P2RX7, CRP, IL6, NLRP3, IL17A
GO:0046427	positive regulation of JAK-STAT cascade	3.66E-05	4	IL10, IL6, CCL5, TYK2
GO:0032496	response to lipopolysaccharide	5.04E-05	5	P2RX7, ACE, IL13, S100A9, S100A8
GO:0070234	positive regulation of T cell apoptotic process	8.96E-05	3	P2RX7, CCL5, PDCD1
GO:0032731	positive regulation of interleukin-1 beta production	1.06E-04	4	P2RX7, IL6, NLRP3, IL17A
GO:0042102	positive regulation of T cell proliferation	1.21E-04	4	CD274, IL6, CCL5, TYK2

GO:0006915	apoptotic process	2.07E-04	7	IRF1, LCN2, NLRP3, PDCD1, S100A9, S100A8, IL17A
GO:0006919	activation of cysteine-type endopeptidase activity involved in apoptotic process	2.13E-04	4	NLRP3, S100A9, F3, S100A8
GO:0042742	defense response to bacterium	4.12E-04	5	IL10, OAS1, LCN2, S100A9, S100A8
GO:0050729	positive regulation of inflammatory response	5.09E-04	4	ACE, NLRP3, S100A9, S100A8
GO:0002639	positive regulation of immunoglobulin production	6.89E-04	3	IL10, IL6, IL13
GO:0060333	interferon-gamma-mediated signaling pathway	6.89E-04	3	IRF1, HLA-C, TYK2
GO:0050832	defense response to fungus	0.0012	3	S100A9, S100A8, IL17A
GO:0007568	aging	0.0012	4	IL10, ACE, SERPING1, TNFRSF1B
GO:0032733	positive regulation of interleukin-10 production	0.0017	3	CD274, IL6, IL13
hsa04657	IL-17 signaling pathway	5.46E-06	6	IL6, IL13, LCN2, S100A9, S100A8, IL17A
hsa05133	Pertussis	5.56E-05	5	IL10, IL6, IRF1, NLRP3, SERPING1
hsa04060	Cytokine-cytokine receptor interaction	1.40E-04	7	IL10, IL6, CCL5, IL13, CCR5, TNFRSF1B, IL17A
hsa04621	NOD-like receptor signaling pathway	1.47E-04	6	P2RX7, IL6, OAS1, CCL5, NLRP3, TYK2
hsa04061	Viral protein interaction with cytokine and cytokine receptor	1.62E-04	5	IL10, IL6, CCL5, CCR5, TNFRSF1B
hsa05171	Coronavirus disease - COVID-19	4.11E-04	6	IL6, ACE, OAS1, VWF, NLRP3, TYK2
hsa05321	Inflammatory bowel disease	7.79E-04	4	IL10, IL6, IL13, IL17A
hsa05164	Influenza A	0.0012	5	IL6, OAS1, CCL5, NLRP3, TYK2
hsa05142	Chagas disease	0.0029	4	IL10, IL6, ACE, CCL5
hsa04625	C-type lectin receptor signaling pathway	0.0030	4	IL10, IL6, IRF1, NLRP3

Supplementary figures

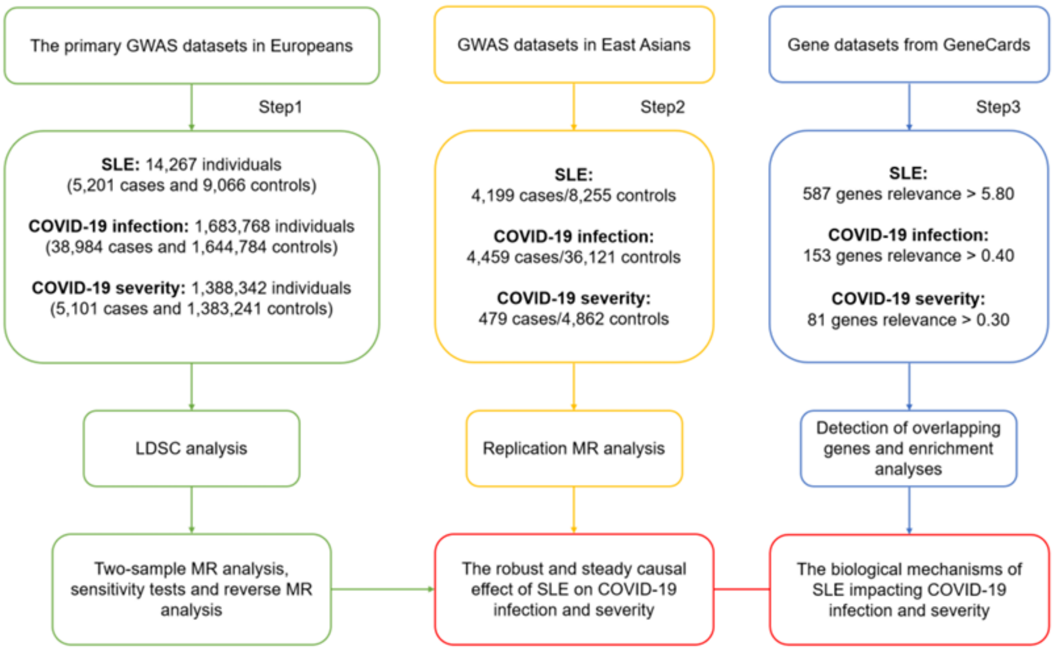

Figure S1. Study Design Overview. Green frames and lines represent analyses conducted using European GWAS datasets, while yellow frames and lines depict analyses utilizing East Asian data. Blue frames and lines indicate the use of GeneCards data, and red frames and lines highlight the primary objectives achieved in each analysis.

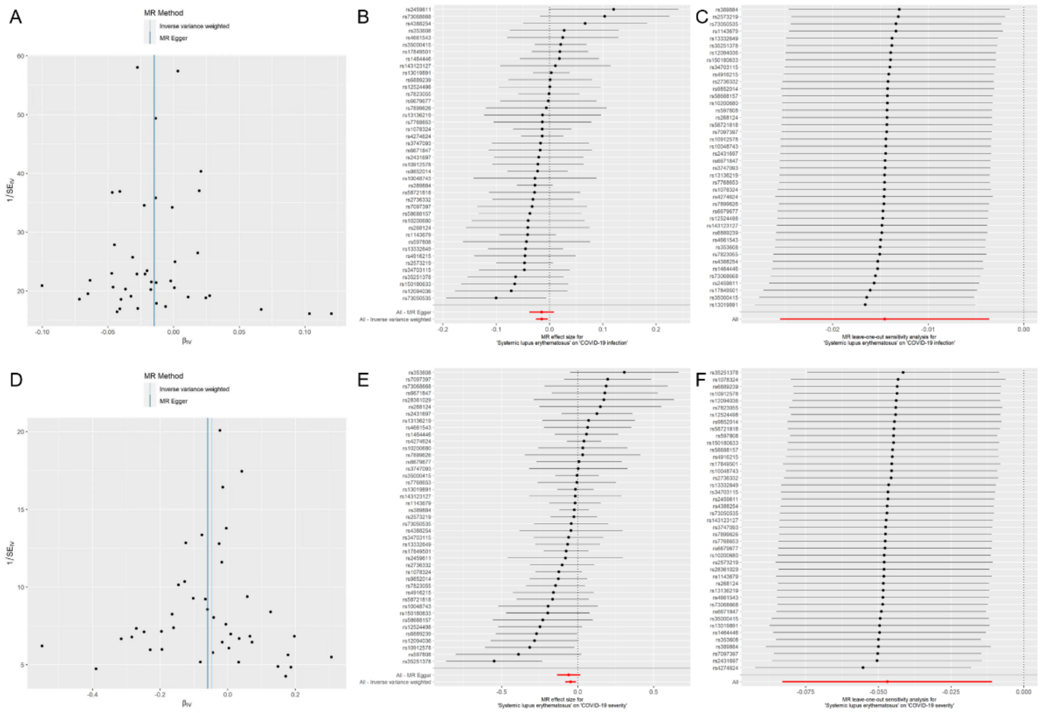

Figure S2. Sensitivity analyses results for the causal effects in Europeans. **Panel A.** funnel plot of SLE on COVID-19 infection. **Panel B.** single SNP plot of SLE on COVID-19 infection. **Panel C.** leave-one-out plot of SLE on COVID-19 infection. **Panel D.** funnel plot of SLE on COVID-19 severity. **Panel E.** single SNP plot of SLE on COVID-19 severity. **Panel F.** leave-one-out plot of SLE on COVID-19 severity.

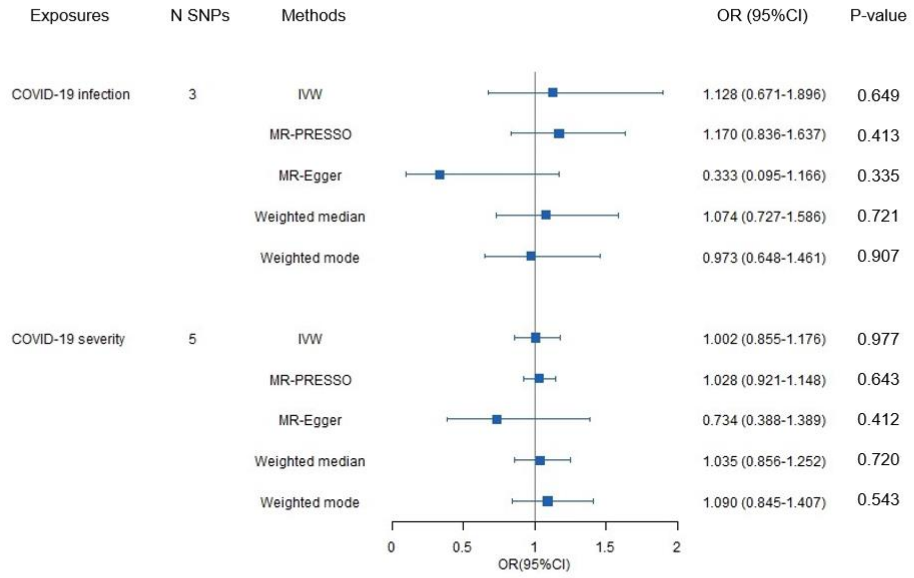

Figure S3. The reverse MR results of COVID-19 infection and severity on SLE in Europeans. A P value was considered statistically significant if < 0.05 (two sides).

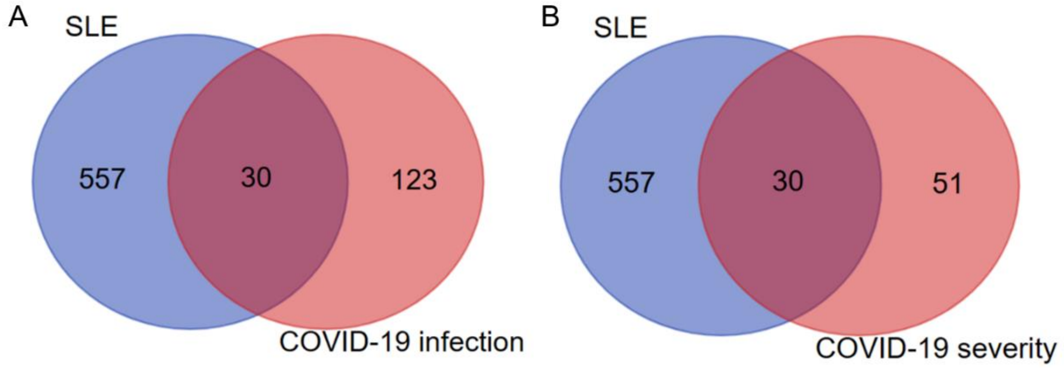

Figure S4. The overlapping genes of SLE with COVID-19 infection and severity respectively. **Panel A.** Venn results between SLE with COVID-19 infection. **Panel B.** Venn results between SLE with COVID-19 severity.
